# Insights and opinions of readers of the *Journal of the Medical Library Association*

**DOI:** 10.5195/jmla.2022.1458

**Published:** 2022-04-01

**Authors:** Katherine G. Akers, JJ Pionke, Ellen Aaronson, Rachel Koenig, Michelle Kraft, Beverly Murphy

**Affiliations:** 1 kgakers@gmail.com, Senior Editor, *JMLA*; 2 pionke@illinois.edu, Proceedings Coeditor, *JMLA*; 3 aaronson.ellen@mayo.edu, Resource Reviews Coeditor, *JMLA*; 4 rakoenig@vcu.edu, Former Editorial Board Member, *JMLA*; 5 kraftm@ccf.org, Virtual Projects Coeditor, *JMLA*; 6 beverly.murphy@duke.edu, In Memoriam Coeditor, *JMLA*

## Abstract

The *Journal of the Medical Library Association (JMLA)* conducted a readership survey in 2020 to gain a deeper understanding of our readers, their reading habits, and their satisfaction with *JMLA*'s content, website functionality, and overall quality. A total of 467 readers responded to the survey, most of whom were librarians/information specialists (85%), worked in an academic (62%) or hospital/health care system (27%) library, and were current Medical Library Association members (80%). Most survey respondents (46%) reported reading *JMLA* articles on a quarterly basis. Over half of respondents (53%) said they used social media to follow new research or publications, with Twitter being the most popular platform. Respondents stated that Original Investigations, Case Reports, Knowledge Syntheses, and Resource Reviews articles were the most enjoyable to read and important to their research and practice. Almost all respondents reported being satisfied or very satisfied (94%) with the *JMLA* website. Some respondents felt that the content of *JMLA* leaned more toward academic librarianship than toward clinical/hospital librarianship and that there were not enough articles on collection management or technical services. These opinions and insights of our readers help keep the *JMLA* editorial team on track toward publishing articles that are of interest and utility to our audience, raising reader awareness of new content, providing a website that is easy to navigate and use, and maintaining our status as the premier journal in health sciences librarianship.

The *Journal of the Medical Library Association* (*JMLA*), in tandem with the Medical Library Association (MLA), periodically conducts readership surveys to gauge how well the journal meets the needs and desires of its readers. However, much time has passed and many changes have occurred since our last readership survey was published in 2013 [1]. These changes include our transition from a largely print-based to an almost fully digital mode of publication, more extensive reliance on providing access to supplemental materials to improve the rigor and reproducibility of research findings, and the use of social media to share and stay abreast of new scholarly works. Therefore, we conducted a readership survey in 2020 to obtain a more current understanding of our readers, their reading habits, and their satisfaction with *JMLA*'s content, website functionality, and overall quality.

The survey was implemented using Qualtrics and consisted of sixteen closed- and open-ended questions (Appendix A). The survey invitation was distributed on July 6, 2020, through MLA email listservs and social media accounts (Twitter, Facebook, LinkedIn), *JMLA*'s Twitter account, and an announcement on the *JMLA* website. Recipients were encouraged to forward the invitation through other communication channels as appropriate. The survey was closed on July 20, 2020.

## RESPONDENT DEMOGRAPHICS

A total of 467 readers responded to the survey. Most respondents (85%) were librarians/information specialists, whereas others were retired (5%), library staff (e.g., assistants, clerks; 4%), academic researchers or educators (3%), health care workers (1%), students (1%), or in other professions (e.g., publishers, vendors; 1%). However, because our survey invitation was sent primarily through professional health sciences librarian communication channels, we suspect that librarians/information specialists may be overrepresented among our survey respondents.

Considering respondents who were librarians/information specialists or library staff, nearly two-thirds worked in an academic library (62%), and nearly one-third worked in a hospital or health care system library (27%). The remaining respondents worked in a public library (4%); federal, state, or military library (3%); or other type of library (e.g., association, research institute, industry; 4%).

Most respondents were current MLA members (80%), whereas others were not current MLA members but had been members in the past (11%) or had never been MLA members (9%).

## READING FREQUENCY

*JMLA* is a quarterly journal, with one issue published every three months. Accordingly, most survey respondents reported reading *JMLA* articles on a quarterly basis (46%). Other respondents reported reading *JMLA* articles once a month (26%), once a year or less frequently (14%), a few times a month (13%), or at least once a week (1%).

## AWARENESS OF ARTICLES

Over half of survey respondents (53%) said they used social media or social networking services to follow new research or publications. Among those who reported using social media or social networking services for this purpose, 35% used Twitter, 21% used Facebook, 20% used LinkedIn, 14% used ResearchGate or Academia.edu, 6% used Mendeley, 6% used Instagram, and <1% used other platforms (e.g., Reddit, Parler, Tumblr, YouTube).

Most respondents reported becoming aware of new *JMLA* articles through quarterly emails containing the table of contents of new issues (80%), professional email listservs (44%), database (e.g., PubMed) searches or alerts (42%), word of mouth (26%), social media (21%), presentations at conferences (19%), receipt of print issues of *JMLA* (11%), or other ways (<1%).

## ACCESSING ARTICLES

Most respondents reported typically accessing the full texts of *JMLA* articles through the *JMLA* website (58%), whereas other respondents said they typically accessed full text of *JMLA* articles through PubMed Central (32%), print issues of *JMLA* (8%), or other routes (2%).

## PREFERENCE FOR ARTICLE TYPES

**Figure 1:**
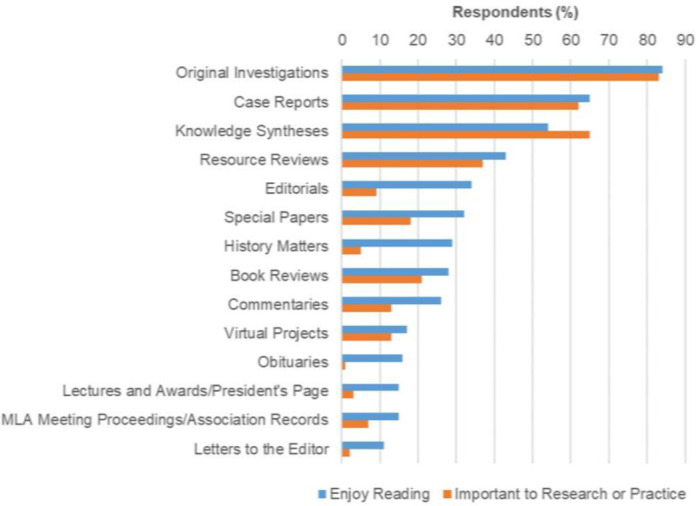
Percentage of respondents who enjoy reading certain article types and find them important to their research or practice

When we asked respondents which article types were most enjoyable to read and most important to their research and practice, respondents most frequently selected Original Investigations (peer-reviewed articles describing research that employs any type of quantitative or qualitative method of analysis), Case Reports (peer-reviewed articles describing the development, implementation, and evaluation of a new service, program, or initiative, typically in a single institution or through a single collaborative effort), Knowledge Syntheses (peer-reviewed review articles, including systematic reviews, scoping reviews, and narrative reviews), and Resource Reviews (critical appraisals of electronic resources, software, web services, and other technology tools that assist health sciences library staff in making collection development and technology implementation decisions) (Figure 1).

When respondents were asked to provide comments about the types of articles that *JMLA* publishes, one major theme was that they felt the content of *JMLA* leans more toward academic librarianship than toward clinical/hospital librarianship. From an editorial perspective, we believe this is a direct result of the fact that most manuscripts submitted to *JMLA* are authored by academic health sciences librarians, perhaps because academic librarians are expected to publish to achieve tenure or promotion or because hospital librarians face different pressures and do not have the time to pursue scholarly publication. Regardless, this sentiment suggests the need for more training and professional support for hospital librarians who wish to engage in scholarly research and publishing. Another theme in respondents' comments was that there were not enough articles on collection management or technical services and too many articles on public services and systematic reviewing. Furthermore, some respondents believe that *JMLA* publishes too much research, whereas others believe that *JMLA* does not publish enough research.

## ARTICLE APPENDIXES AND UNDERLYING DATA

The rigor and reproducibility of research can be enhanced, in part, by providing access to supporting research materials, including data collection instruments, datasets, and code [2]. *JMLA* has long required survey instruments to be submitted and published alongside articles as appendixes, and we often publish additional supplemental materials such as evaluation rubrics, extra tables and figures, and supporting text. Furthermore, *JMLA* established a data sharing policy in 2019 that requires authors to include a data availability statement in their published article describing how and where the data underlying their results can be accessed [3]. When we asked respondents how important it was for them to be able to access the appendixes and/or data associated with *JMLA* articles, many found it moderately important (33%), somewhat important (32%), or very important (29%), whereas only some (6%) indicated that it was not important to be able to access the appendixes and/or data associated with *JMLA* articles. Nearly half of respondents (49%) said they accessed these materials sometimes, 29% accessed them rarely, 14% accessed them often, and 8% never accessed them.

## JOURNAL WEBSITE

*JMLA* moved to a new publisher (University Library System, University of Pittsburgh) and new submission and publishing platform (Open Journal Systems [OJS] 2) in 2016. Most respondents reported being satisfied (81%) or very satisfied (13%) with the *JMLA* website, although some were dissatisfied (5%) or very dissatisfied (1%). The most common complaints about the *JMLA* website were that its search function does not perform well, that too many clicks are required to arrive at a desired article, that it is difficult to navigate between articles, and that the visual appearance of the website is outdated. We hope that at least some of these concerns have been addressed through an upgrade to OJS 3 earlier this year.

## JOURNAL QUALITY

When asked about the perceived quality of *JMLA* compared with other peer-reviewed journals for health sciences librarians and information professionals, 55% of respondents found *JMLA* to be of similar quality, 44% of higher quality, and 1% of lower quality.

## CONCLUSIONS

The opinions and insights of our readers help keep the *JMLA* editorial team on track toward publishing articles that are of interest and utility to our audience, raising reader awareness of new content, ensuring that articles and accompanying materials are accessible to readers, providing a website that is easy to navigate and use, and maintaining our status as the premier journal in health sciences librarianship. Areas for improvement include soliciting more manuscripts relevant to clinical and hospital librarianship, collection management, and technical services and improving the user experience of the *JMLA* website.

## Data Availability

Raw anonymized survey data are available in the Open Science Framework at https://osf.io/ck467/.

## SUPPLEMENTAL FILES

**Appendix A.** 2020 *JMLA* Readership Survey

## References

[1] Starr S. Journal of the Medical Library Association readership survey. J Med Libr Assoc. 2013 Jul;101(3):167. DOI: 10.3163/1536-5050.101.3.001. 23930083 PMC3738072

[2] Sayre F, Riegelman A. The reproducibility crisis and academic libraries. Coll Res Libr. 2018;79(1):2. DOI: 10.5860/crl.79.1.2.

[3] Akers KG, Read KB, Amos L, Federer LM, Logan A, Plutchak TS. Announcing the Journal of the Medical Library Association's data sharing policy. J Med Libr Assoc. 2019 Oct;107(4):468–71. DOI: 10.5195/jmla.2019.801. 31607804 PMC6774558

