# Supplementary material for: Insights and opinions of readers of the Journal of the Medical Library Association
Source: J Med Libr Assoc. 2022 Apr 1;110(2):156–8. doi: 10.5195/jmla.2022.1458 (PMC9014915; doi:10.5195/jmla.2022.1458)
Supplement: Supplementary file 1 — Appendix A. 2020 JMLA Readership Survey [file jmla-110-2-156-s01.pdf]

Q1 What is your primary role/profession? [choose one]

Answered: 467   Skipped: 1

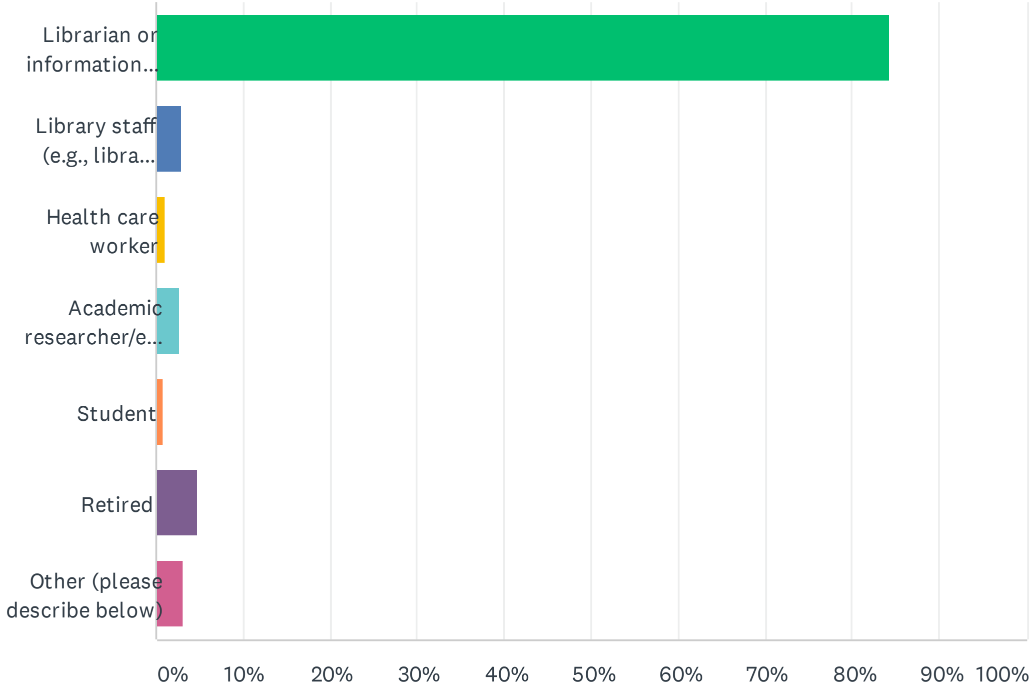

ANSWER CHOICES	RESPONSES	
Librarian or information professional	84.37%	394
Library staff (e.g., library assistant, library clerk)	3.00%	14
Health care worker	1.07%	5
Academic researcher/educator	2.78%	13
Student	0.86%	4
Retired	4.71%	22
Other (please describe below)	3.21%	15
TOTAL		467

#	OTHER (PLEASE DESCRIBE BELOW)	DATE
1	Emergency First Responder	7/20/2020 11:30 AM
2	Peer Support Specialist	7/16/2020 2:31 PM
3	Sales person for publisher	7/16/2020 9:47 AM
4	Publisher	7/10/2020 5:34 AM
5	Archivist	7/7/2020 4:39 PM
6	Vendor	7/7/2020 3:39 PM
7	IS Professor	7/7/2020 3:35 PM
8	Library Director	7/7/2020 3:35 PM
9	Retired and academic researcher	7/7/2020 2:30 PM
10	Academic/Health Educator/Resource Librarian	7/7/2020 12:43 PM
11	Library Manager	7/7/2020 10:48 AM
12	I am retired, but as an emerita, I continue teaching medical students one specific course.	7/7/2020 10:21 AM
13	Library Technician	7/7/2020 7:06 AM
14	librarian and researcher/educator	7/6/2020 7:31 PM
15	Medical journal editor	7/6/2020 7:15 PM

Q2 Which of the following best describes your institution? [choose one]

Answered: 409 Skipped: 59

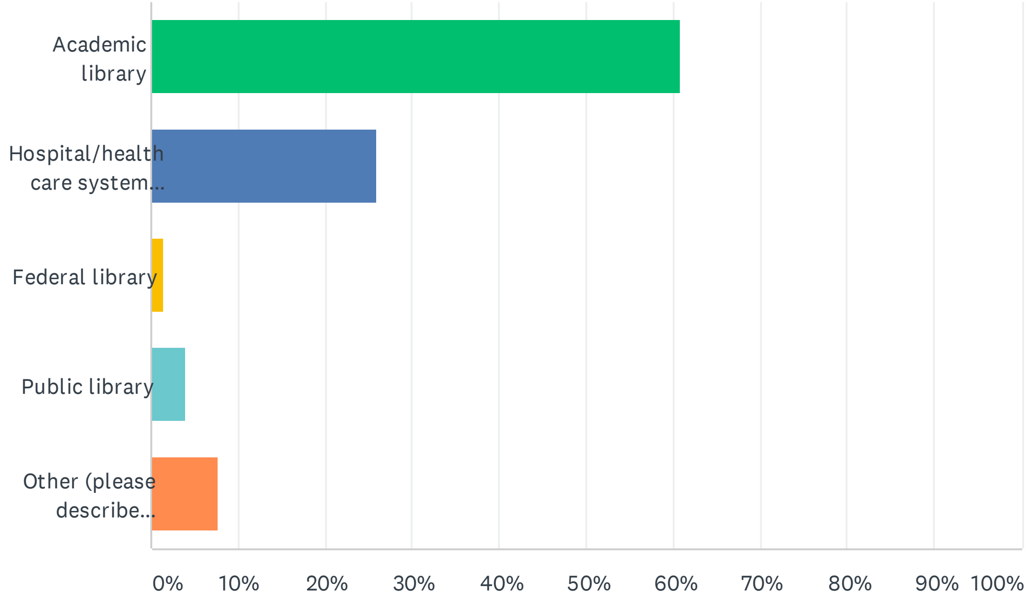

ANSWER CHOICES	RESPONSES	
Academic library	60.88%	249
Hospital/health care system library	25.92%	106
Federal library	1.47%	6
Public library	3.91%	16
Other (please describe below):	7.82%	32
TOTAL		409

#	OTHER (PLEASE DESCRIBE BELOW):	DATE
1	Medical School Library	7/20/2020 2:38 PM
2	consulting firm	7/20/2020 10:19 AM
3	health policy and evidence organization	7/17/2020 12:42 PM
4	State Library	7/16/2020 9:56 AM
5	Cancer research institute and hospital in a university system	7/16/2020 9:52 AM
6	State Library	7/16/2020 9:47 AM
7	Hosptial/health care system library which is Federal	7/16/2020 9:43 AM
8	research institute	7/16/2020 9:36 AM
9	Corporate library	7/16/2020 9:32 AM
10	Association/Medical Society Library	7/13/2020 3:55 PM
11	Medical School	7/10/2020 2:36 PM
12	Pharma industry	7/10/2020 10:43 AM
13	tribal library	7/9/2020 11:59 AM
14	Academic Health Library	7/9/2020 8:46 AM
15	military teaching hospital	7/8/2020 10:34 PM
16	Both academic and health care system	7/8/2020 1:33 PM
17	Informationist freelancer with science (PhD) background	7/8/2020 3:27 AM
18	Vendor	7/7/2020 3:22 PM
19	Publishing company (for now)	7/7/2020 2:39 PM
20	Publishing	7/7/2020 1:53 PM
21	Medical Research Center Library	7/7/2020 12:32 PM
22	Library Consortium	7/7/2020 12:10 PM
23	Academic Medical Center	7/7/2020 11:55 AM
24	Teaching Hospital	7/7/2020 11:48 AM
25	Grant-funded health research organization	7/7/2020 11:46 AM
26	hospital library but also federal / military	7/7/2020 11:28 AM
27	association library	7/7/2020 10:36 AM
28	Non-profit association	7/7/2020 10:25 AM
29	Hospital/research	7/7/2020 9:11 AM
30	Government reseach analysis team	7/7/2020 6:45 AM
31	Special	7/7/2020 4:54 AM
32	Scientific instititution	7/7/2020 12:07 AM

Q3 Are you a member of the Medical Library Association (MLA)? [choose one]

Answered: 458 Skipped: 10

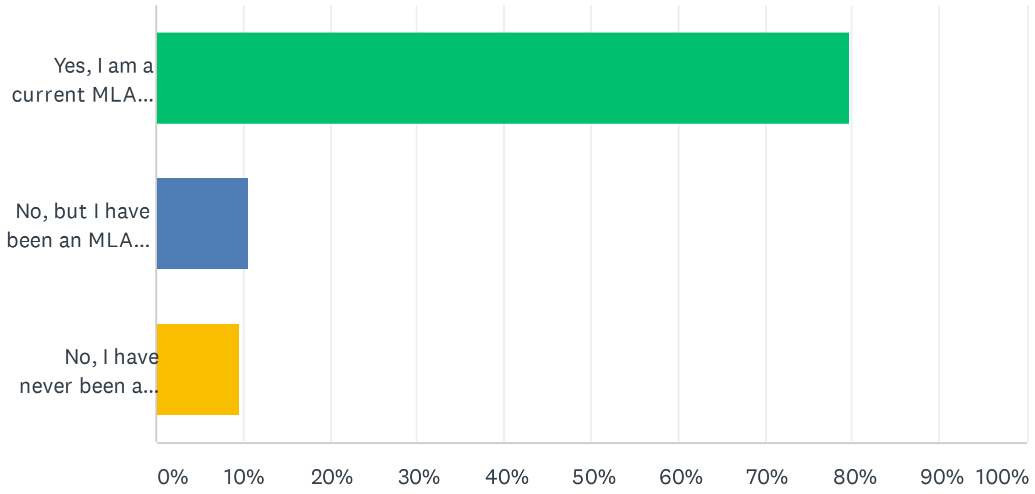

ANSWER CHOICES	RESPONSES	
Yes, I am a current MLA member	79.69%	365
No, but I have been an MLA member in the past	10.70%	49
No, I have never been an MLA member	9.61%	44
TOTAL		458

Q4 How frequently do you read JMLA articles?

Answered: 447 Skipped: 21

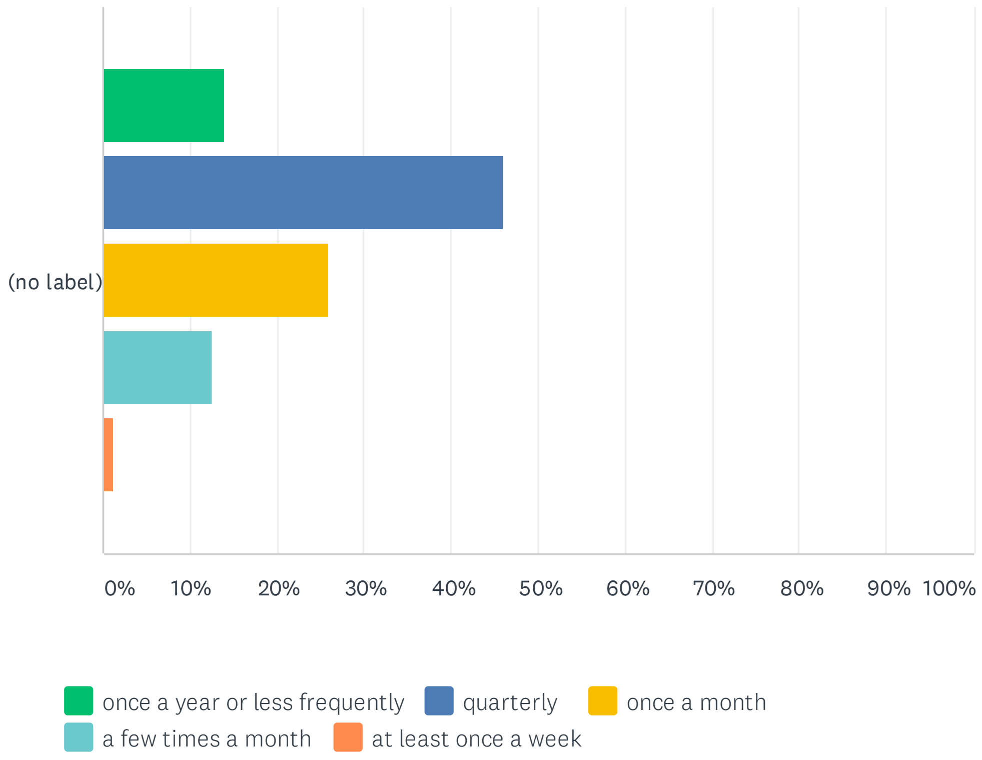

	ONCE A YEAR OR LESS FREQUENTLY	QUARTERLY	ONCE A MONTH	A FEW TIMES A MONTH	AT LEAST ONCE A WEEK	TOTAL	WEIGHTED AVERAGE
(no label)	14.09% 63	46.09% 206	25.95% 116	12.53% 56	1.34% 6	447	2.41

Q5 Which social media platforms or social networking services do you use to follow new research or publications in your field? [choose all that apply]

Answered: 458 Skipped: 10

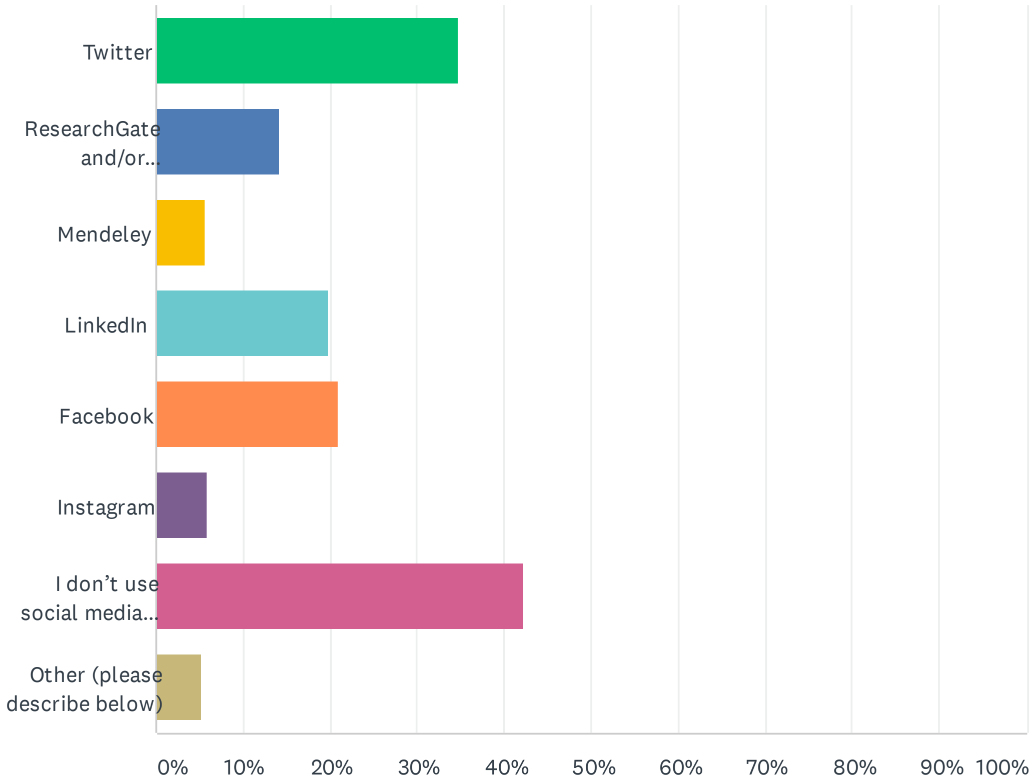

ANSWER CHOICES	RESPONSES	
Twitter	34.72%	159
ResearchGate and/or Academia.edu	14.19%	65
Mendeley	5.68%	26
LinkedIn	19.87%	91
Facebook	20.96%	96
Instagram	5.90%	27
I don't use social media or social networking services to follow new research or publications	42.36%	194
Other (please describe below)	5.24%	24
Total Respondents: 458		

#	OTHER (PLEASE DESCRIBE BELOW)	DATE
1	Reddit	7/16/2020 2:14 PM
2	RSS Feeds to Library and leadership websites and journals	7/16/2020 11:19 AM
3	Academia	7/16/2020 11:12 AM
4	Listservs and email alerts. Interested in Twitter though.	7/16/2020 9:47 AM
5	recommendations provided in different email addresses	7/16/2020 9:43 AM
6	Parler	7/16/2020 9:34 AM
7	Listservs	7/13/2020 10:05 AM
8	E-mail alerts from various publishers	7/10/2020 7:57 AM
9	ORCID, web of science reports etc.	7/9/2020 1:21 PM
10	email alerts	7/9/2020 12:01 PM
11	RSS Feed Reader, DOAJ	7/9/2020 11:48 AM
12	Email	7/8/2020 4:29 PM
13	RSS feeds or email alerts	7/8/2020 2:08 PM
14	BrowZine, etc.	7/8/2020 11:22 AM
15	PubMed, WoS, Scopus, SciFinder	7/8/2020 3:29 AM
16	Read by QxMD, journal alerts, search alerts	7/7/2020 4:28 PM
17	listservs	7/7/2020 3:41 PM
18	email	7/7/2020 3:00 PM
19	Searching in databases as needed	7/7/2020 12:35 PM
20	communication lists	7/7/2020 11:56 AM
21	Tumblr and YouTube	7/7/2020 10:55 AM
22	Google Scholar recommendations	7/7/2020 10:44 AM
23	I follow people on those platforms and those people post about articles, I don't really use them to follow pubs.	7/6/2020 11:54 PM
24	I use PubMed canned searches on topics of continuing interest	7/6/2020 7:17 PM

Q6 How do you typically become aware of new JMLA articles? [choose all that apply]

Answered: 456 Skipped: 12

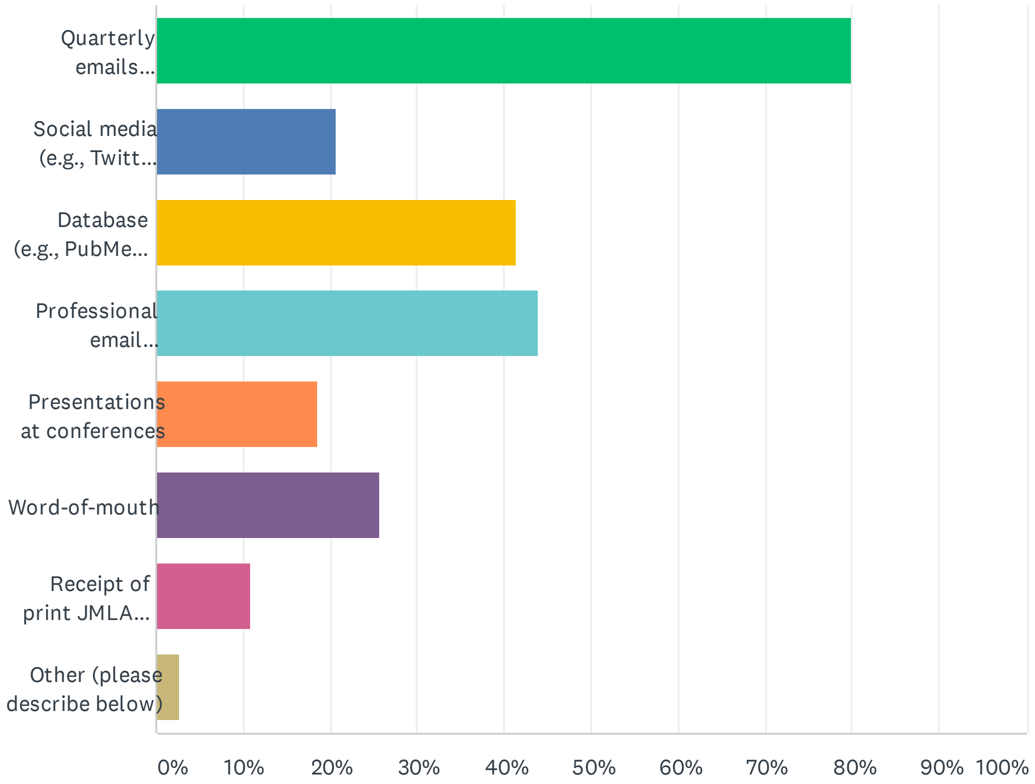

ANSWER CHOICES	RESPONSES	
Quarterly emails containing the table of contents of new JMLA issues	79.82%	364
Social media (e.g., Twitter, Facebook) posts about JMLA articles or issues	20.61%	94
Database (e.g., PubMed) searches	41.45%	189
Professional email discussion lists	43.86%	200
Presentations at conferences	18.64%	85
Word-of-mouth	25.66%	117
Receipt of print JMLA issues	10.96%	50
Other (please describe below)	2.63%	12
Total Respondents: 456		

#	OTHER (PLEASE DESCRIBE BELOW)	DATE
1	If it comes up in one of my custom PubMed alerts	7/17/2020 12:46 PM
2	access to JMLA website	7/16/2020 8:27 PM
3	PubMed results	7/16/2020 10:51 AM
4	The email MLA sends out when there's a new issue	7/13/2020 9:14 AM
5	The MLA Connect newsletter and checking the MLA site.	7/8/2020 10:31 AM
6	Via bibliography	7/7/2020 12:44 PM
7	Browzine	7/7/2020 10:24 AM
8	I just check in periodically	7/7/2020 9:19 AM
9	Read by QxMD	7/7/2020 8:01 AM
10	Google search	7/7/2020 8:01 AM
11	physical journal in the snail mail	7/7/2020 2:35 AM
12	journal club in workplace	7/6/2020 7:31 PM

Q7 How do you typically access the full texts of JMLA articles? [choose one]

Answered: 453 Skipped: 15

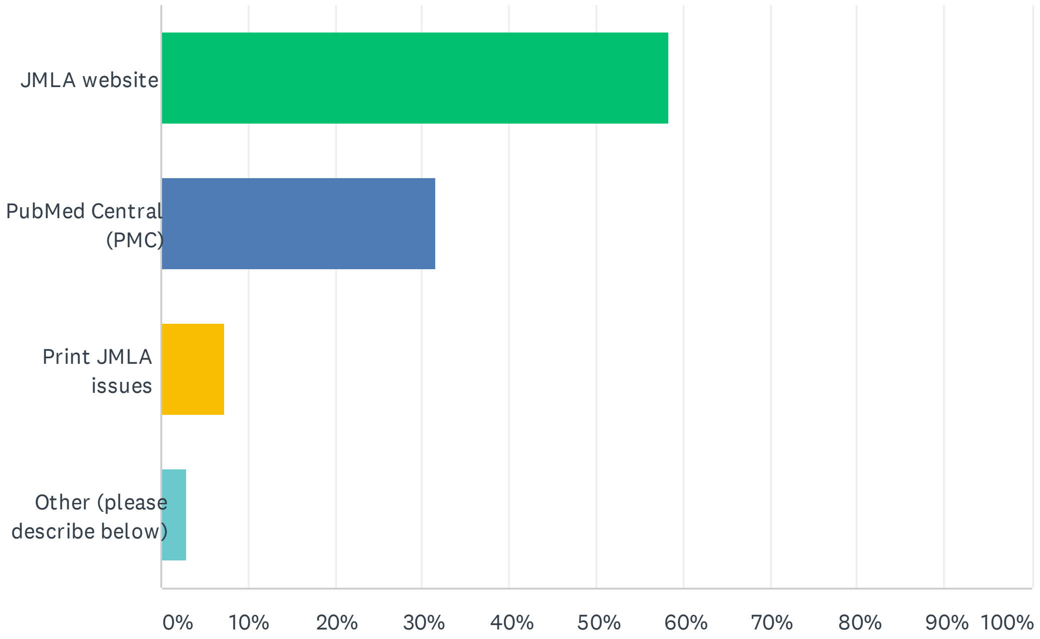

ANSWER CHOICES	RESPONSES	
JMLA website	58.28%	264
PubMed Central (PMC)	31.57%	143
Print JMLA issues	7.28%	33
Other (please describe below)	2.87%	13
TOTAL		453

#	OTHER (PLEASE DESCRIBE BELOW)	DATE
1	Google and Google scholar	7/16/2020 6:42 PM
2	Other databases	7/16/2020 10:28 AM
3	Periodic sharing from friends	7/9/2020 1:07 AM
4	use all three	7/8/2020 10:36 PM
5	BrowZine	7/8/2020 7:31 AM
6	use the toc when it arrives to link to article	7/7/2020 3:37 PM
7	Our own subscription	7/7/2020 3:36 PM
8	I always accessed all article when my print issues came in. Now that I'm not doing print due to pricing, I rarely access the full text; even then, when the quarterly email comes in, I might open access to one or two articles with an interesting title.	7/7/2020 2:51 PM
9	It depends if I'm reading JMLA or came across the article in a PubMed search.	7/7/2020 2:40 PM
10	EBSCO Host TOC	7/7/2020 12:41 PM
11	Varies	7/7/2020 11:49 AM
12	Through our library's link resolver	7/7/2020 10:44 AM
13	Links from LISTA	7/7/2020 10:15 AM

Q8 Which of the following types of JMLA articles do you most enjoy reading [choose all that apply]

Answered: 438   Skipped: 30

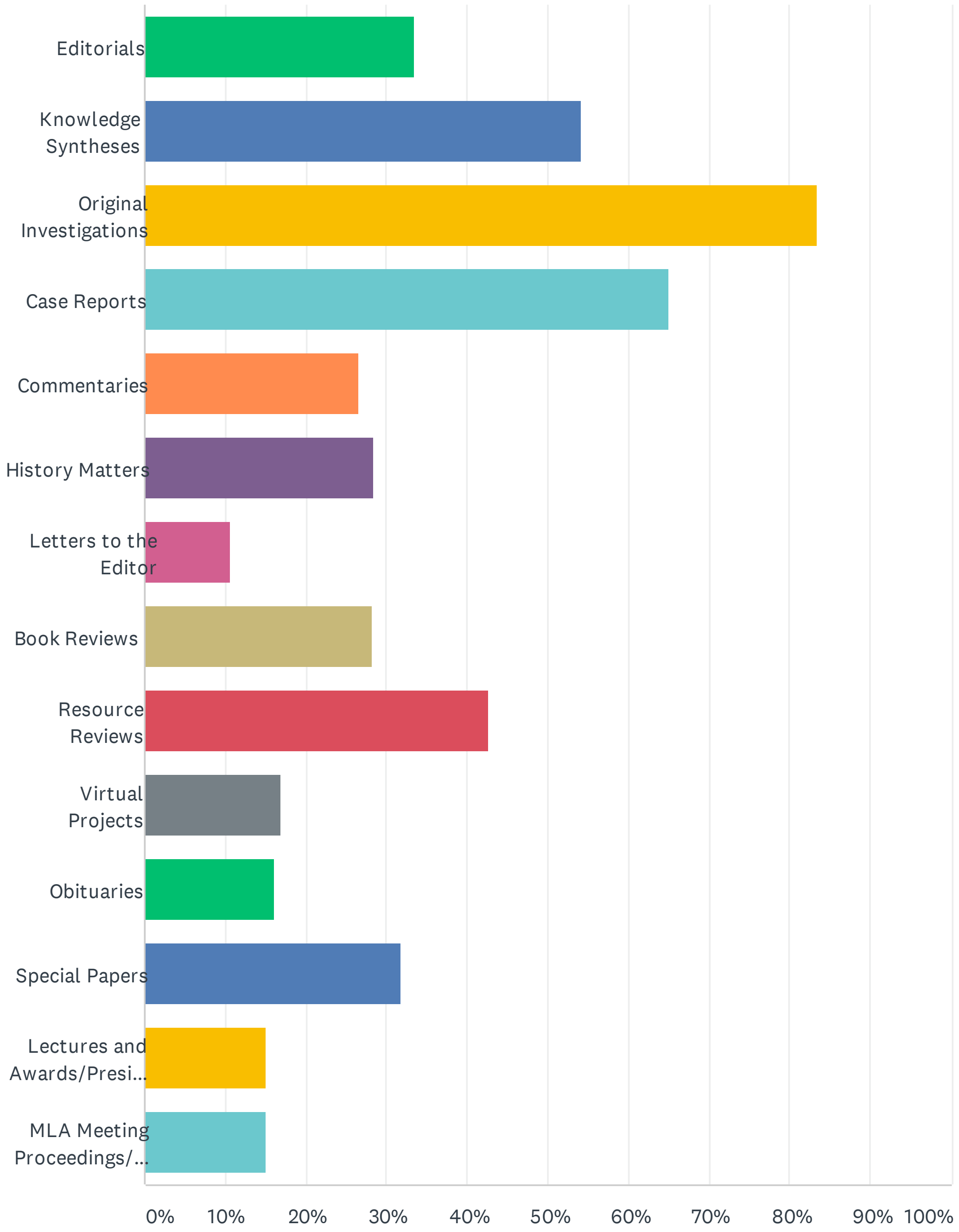

ANSWER CHOICES	RESPONSES	
Editorials	33.56%	147
Knowledge Syntheses	54.11%	237
Original Investigations	83.56%	366
Case Reports	65.07%	285
Commentaries	26.48%	116
History Matters	28.54%	125
Letters to the Editor	10.73%	47
Book Reviews	28.31%	124
Resource Reviews	42.69%	187
Virtual Projects	16.89%	74
Obituaries	16.21%	71
Special Papers	31.74%	139
Lectures and Awards/President’s Page	15.07%	66
MLA Meeting Proceedings/Association Records	15.07%	66
Total Respondents: 438		

Q9 Which of the following types of JMLA articles are most important to your research or practice [choose all that apply]

Answered: 429 Skipped: 39

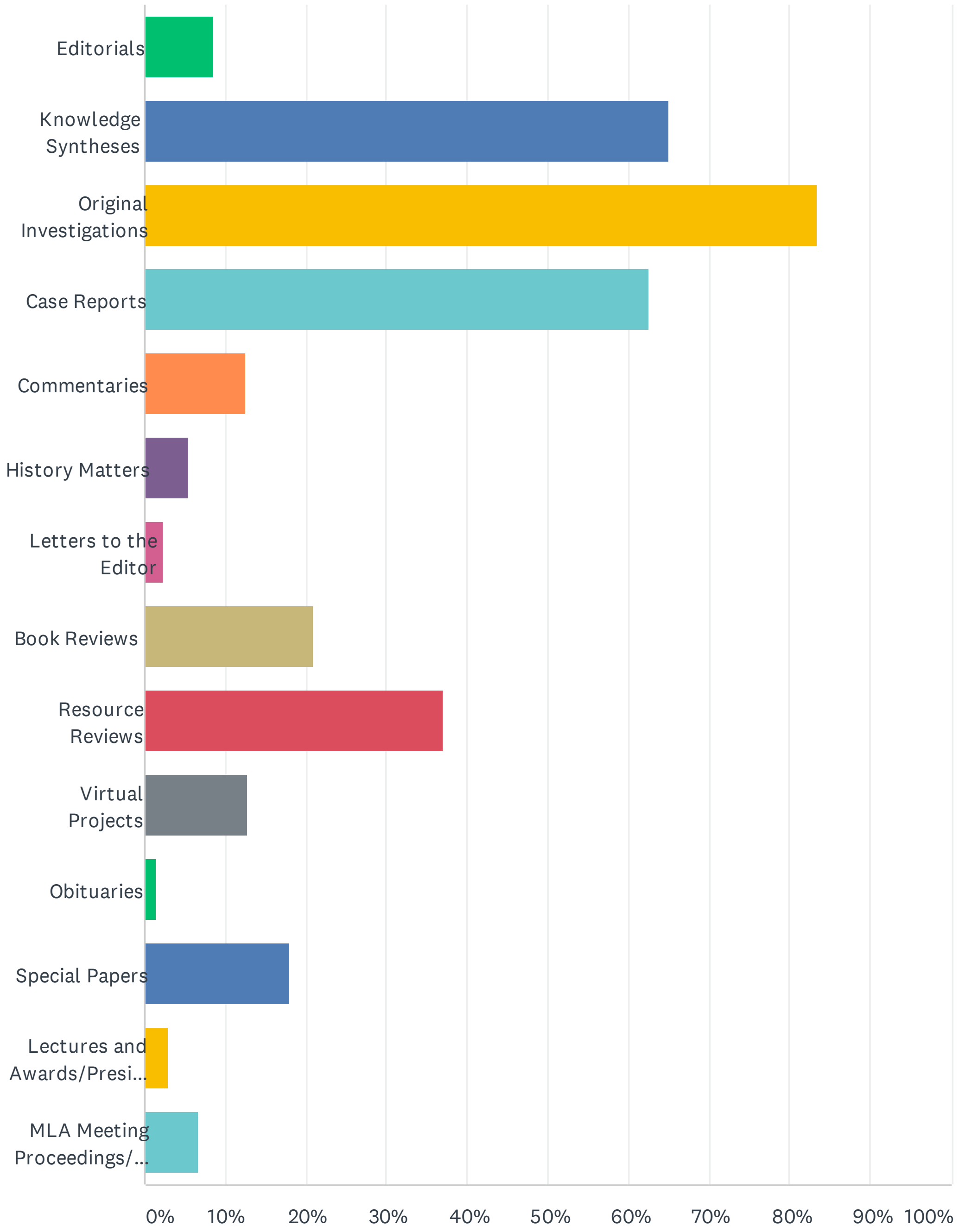

ANSWER CHOICES	RESPONSES	
Editorials	8.62%	37
Knowledge Syntheses	65.03%	279
Original Investigations	83.45%	358
Case Reports	62.47%	268
Commentaries	12.59%	54
History Matters	5.36%	23
Letters to the Editor	2.33%	10
Book Reviews	20.98%	90
Resource Reviews	37.06%	159
Virtual Projects	12.82%	55
Obituaries	1.40%	6
Special Papers	17.95%	77
Lectures and Awards/President’s Page	3.03%	13
MLA Meeting Proceedings/Association Records	6.76%	29
Total Respondents: 429		

Q10 Please provide any comments about the types of articles that JMLA publishes.

Answered: 55 Skipped: 413

#	RESPONSES	DATE
1	I honestly enjoy them all. I don't have time to read all of them right when they are published, but I've found so much helpful content at the point of need, so even at a later date the articles are helpful to me. In terms of data, I find it most helpful when I can find the survey instruments that are used in studies - because often I can fashion mind from theirs (and give proper attribution, of course).	7/20/2020 4:26 PM
2	They are timely, current and to the point. Very useful work done by medical librarians.	7/19/2020 8:30 PM
3	I like review articles, articles about librarian projects, new resources, librarians working in other countries	7/16/2020 6:07 PM
4	Most value to me are the ones that I can get new ideas from and then apply	7/16/2020 11:34 AM
5	They are mostly focused on university/academic institutions that bear zero resemblance to what hospital librarians do or need. I don't have "staff"; I have ME. MLA does not address this and while I hope that the influx of hospital librarians recently helps, it's an institutional CHOICE to ignore over half your membership. I don't know if it is half, but it's certainly a significant percentage, those of us who are left, that is, another issue MLA had pretty much ignored; heaven knows there's never been an public advocacy or press release regarding the loss of hospital librarian positions. We feed you and the NLM. Y'all need to recognize and appreciate that... If you aren't getting hospital librarian research, foster it, all the way to publication. Work with NLM (hello?) from grant to proof. Then you/we will have articles that are pertinent to me.	7/16/2020 9:46 AM
6	JMLA almost never contains any content interesting to my field of practice. I rely on other library journals.	7/16/2020 9:35 AM
7	They are very specialized and focused in a certain topic. They are relevant of that area of investigation. Thanks.	7/16/2020 9:17 AM
8	N/A	7/14/2020 8:47 AM
9	It's heavy on the academic site and I struggle to find value or application in the content as a hospital librarian.	7/13/2020 9:26 PM
10	informative	7/9/2020 12:04 PM
11	I would like to see more on Justice, Equity, Diversity, and Inclusion work. This should really just be a standard of the profession at this point and I don't see it represented as much in this journal, that is why I read it less.	7/9/2020 11:57 AM
12	Not exciting	7/9/2020 1:13 AM
13	Frankly, I read JMLA a lot more often when I received a print copy. Lately I have only read it when something peaked my interest or if I am doing research of some sort	7/8/2020 2:32 PM
14	A variety is essential!	7/8/2020 2:10 PM
15	A good variety for the many areas of readership	7/8/2020 11:33 AM
16	Love the Jmla and look forward to it.	7/7/2020 8:06 PM
17	I appreciate knowing which articles receive review status for AHIP credit. Overall, the article topics are relevant and timely. Is it time to leave the "issue" concept and publish when available? On second thought, never mind.	7/7/2020 5:06 PM
18	I like the structured abstracts	7/7/2020 3:39 PM
19	Highly relevant to the profession, but not necessarily meeting my immediate needs while I'm in the profession.	7/7/2020 2:54 PM
20	There seem to be fewer research (RCTs, large surveys, and other empirical methods), articles on the value of health science libraries , services and collections, than there were 5 years or so ago. Is this by design or are our researcher/authors just doing less of this?	7/7/2020 2:40 PM
21	I often wish that there were more practical "how to" articles.	7/7/2020 2:35 PM
22	I deal with electronic resources. It seems that many of the articles and webinars have moved away from that and there is a lot more on public services. There seems to be less content that is relevant to my needs.	7/7/2020 2:05 PM

23	As far as case reports, I'm guessing this is also referring to Case Studies. I prefer reading about best practices in library settings similar to mine.	7/7/2020 1:42 PM
24	Hard to define just one column as valuable, rather I read what interests me	7/7/2020 12:54 PM
25	All are important	7/7/2020 12:47 PM
26	Would like to see articles including explanation of number of library staff needed for project/automation/service/resource access to succeed to help hospital librarians justify full-time positions for more than one librarian.	7/7/2020 12:39 PM
27	quality has improved over the years	7/7/2020 11:59 AM
28	I love the resource reviews, commentaries, editorials, book reviews, etc. … I honestly don't have a lot of time to read the in-depth research studies published by librarians.	7/7/2020 10:53 AM
29	You rarely have any material about technical services, and SO MUCH about systematic reviews (although it's better than it used to be.) I'd love to read more articles having to deal with ILL, cataloging, and other tech services topics as they relate specifically to medical librarianship.	7/7/2020 10:42 AM
30	I miss the fact that there are not much advanced research articles for advanced medical library professionals.	7/7/2020 10:40 AM
31	I especially appreciate "how to" or "how we did it" type articles.	7/7/2020 10:33 AM
32	I really enjoy the projects on "mapping the literature"	7/7/2020 10:23 AM
33	I've been a JMLA reader for decades and have seen some real ups and downs in terms of editorial control. Lately things have been better! The fact that MLA does not publish conference proceedings means that JMLA has a very important role to play in disseminating the research work relevant to medical librarianship. JMLA's standards need to remain high or we'll turn into the Journal of Hospital Librarianship!	7/7/2020 10:14 AM
34	Mostly innovative	7/7/2020 9:40 AM
35	I am looking more for the application of processes in the healthcare setting/hospital setting. Many times the research oriented pieces become too technical to make sense of how to apply the knowledge.	7/7/2020 9:38 AM
36	Find the articles not really relevant to the hospital library setting and too many topics that aren't applicable in multi-site libraries.	7/7/2020 9:12 AM
37	n/a	7/7/2020 8:50 AM
38	More research, both qualitative and quantitative is needed in this profession. Those are the articles of interest for further research and for showing information science and librarianship as a serious, scholarly, and actionable profession, contributing to its growth and ability to serve its communities.	7/7/2020 8:49 AM
39	It would be great to have more theory-based papers.	7/7/2020 7:48 AM
40	I find articles in JMLA much less informative than articles in other journals. Articles about technology are narrowly focused and repetitive.	7/7/2020 7:31 AM
41	Really enjoyed the recent piece about Outlander	7/7/2020 7:22 AM
42	The quality is usually great, but I would love to see more items about clinical librarianship.	7/7/2020 7:19 AM
43	I don't find much that I really need to read.	7/7/2020 7:18 AM
44	Most of them are way too academic to have relevance in a busy clinical setting.	7/7/2020 5:58 AM
45	All are great as peer-reviewed.	7/7/2020 5:49 AM
46	They are the referent. The route to follow.	7/7/2020 5:10 AM
47	special papers are important and it should has comprehensive information	7/7/2020 3:20 AM
48	I read most of the original articles and knowledge syntheses on PubMed Central as a result of searching on topics. I read the other items on the journal website when the new issue is announced.	7/6/2020 11:58 PM

49	as a retired practitioner and slis professor, I enjoy seeing what my former colleagues and former students are writing	7/6/2020 9:12 PM
50	Trust worthy.	7/6/2020 9:00 PM
51	Articles about filters, database search techniques, and process of systematic reviewing are of great interest.	7/6/2020 8:51 PM
52	often not very relevant to my area of practice	7/6/2020 8:34 PM
53	Too many articles that are too research-heavy. I'd prefer more case studies, summaries of outreach projects, problem-solving ideas, overviews from different voices.	7/6/2020 7:53 PM
54	Case reports are often the most useful and relevant to my work.	7/6/2020 7:20 PM
55	A lot of it is not pertinent to my current position	7/6/2020 7:16 PM

Q11 How important is it for you to be able to access the appendixes and/or data associated with JMLA articles?

Answered: 438 Skipped: 30

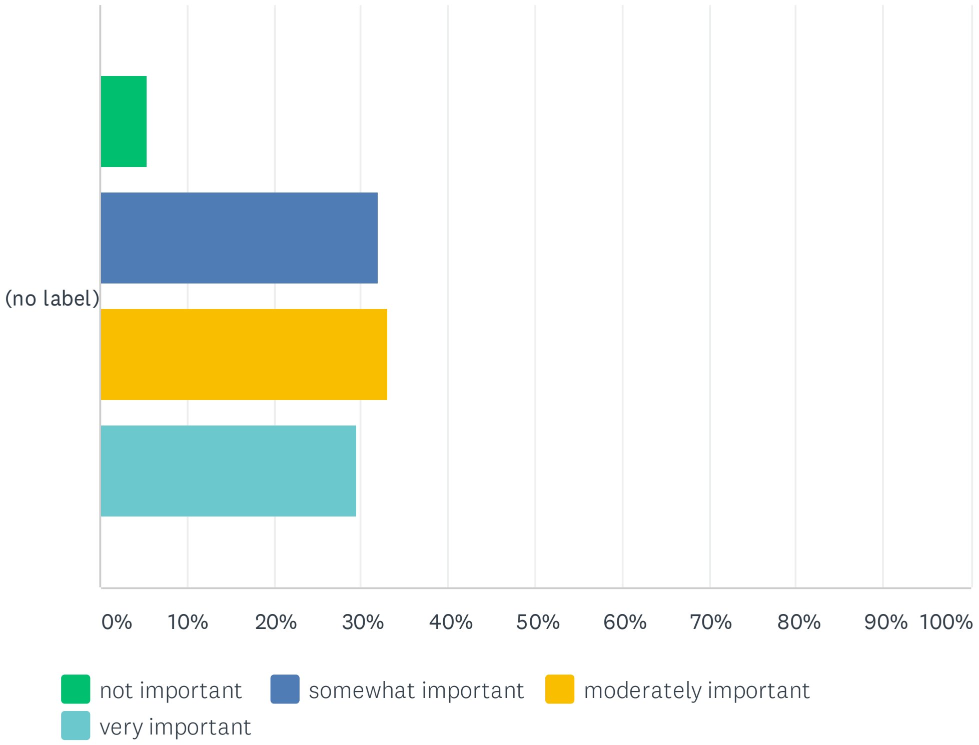

	NOT IMPORTANT	SOMEWHAT IMPORTANT	MODERATELY IMPORTANT	VERY IMPORTANT	TOTAL	WEIGHTED AVERAGE
(no label)	5.48%<br>24	31.96%<br>140	33.11%<br>145	29.45%<br>129	438	2.87

Q12 How frequently do you access the appendixes and/or data associated with JMLA articles?

Answered: 435   Skipped: 33

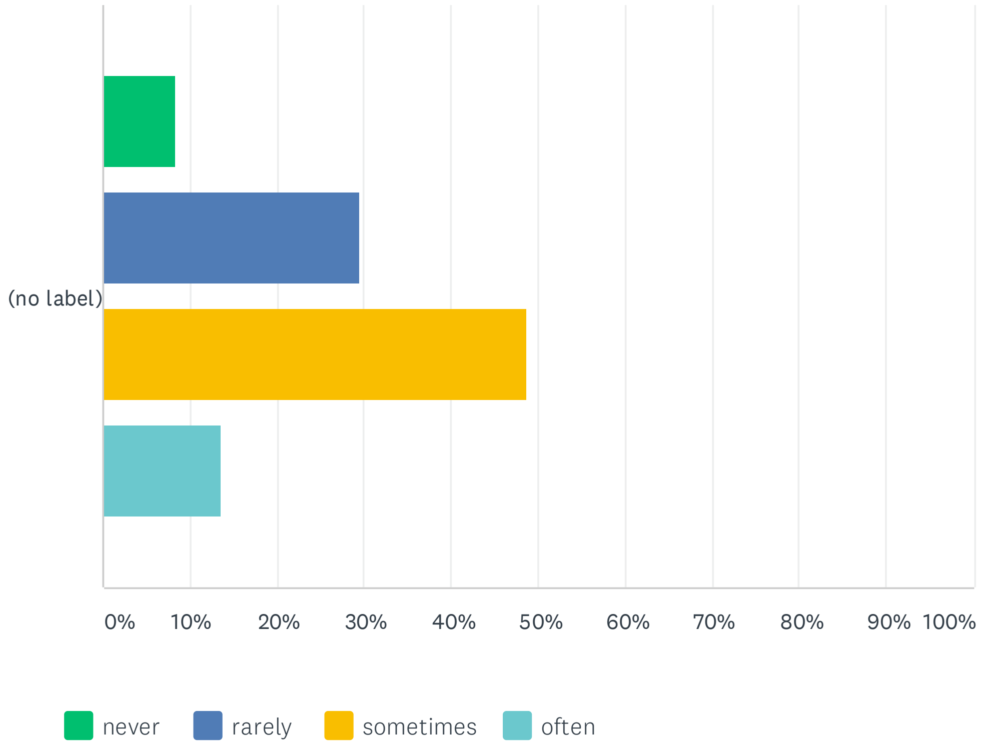

	NEVER	RARELY	SOMETIMES	OFTEN	TOTAL	WEIGHTED AVERAGE
(no label)	8.28%<br>36	29.43%<br>128	48.74%<br>212	13.56%<br>59	435	2.68

Q13 As a reader, how satisfied are you with the JMLA website?

Answered: 431 Skipped: 37

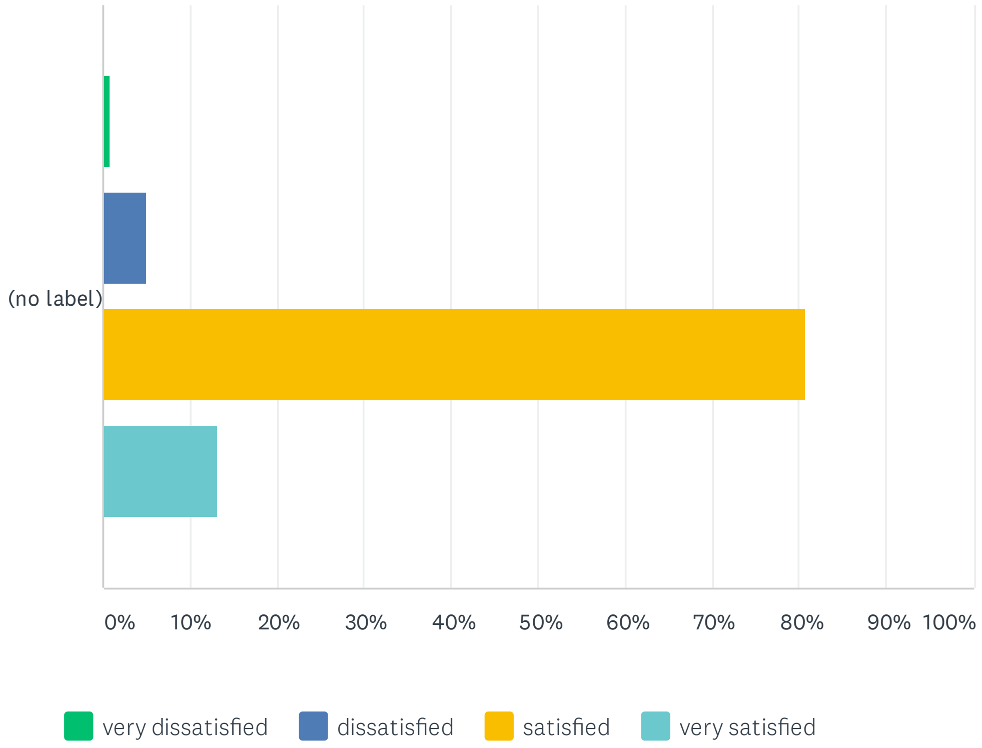

	VERY DISSATISFIED	DISSATISFIED	SATISFIED	VERY SATISFIED	TOTAL	WEIGHTED AVERAGE
(no label)	0.93%<br>4	5.10%<br>22	80.74%<br>348	13.23%<br>57	431	3.06

Q14 Please provide any comments about the JMLA website.

Answered: 59 Skipped: 409

#	RESPONSES	DATE
1	None	7/20/2020 4:26 PM
2	The archives are very helpful.	7/20/2020 11:19 AM
3	It's ok, just not as easy to find/use as the PMC archives	7/19/2020 9:45 PM
4	It is very useful.	7/19/2020 8:30 PM
5	It is a fast and an effective website	7/18/2020 4:19 AM
6	JMLA is a valuable journal, and I regularly review the TOC of each new issue for relevance and often refer articles to colleagues	7/16/2020 9:49 PM
7	the search function is not very good	7/16/2020 6:07 PM
8	It's a little tedious navigating between issues.	7/16/2020 10:15 AM
9	The site is fine, but I admit that I more often access things via PMC.	7/16/2020 10:04 AM
10	It's completely hidden. Not aware of it at all. That may be me, but I don't see it promoted much at all. Why y'all don't want to promote your journal, which is well done and well thought of, escapes me, but you need a serious marketer.	7/16/2020 9:46 AM
11	It is a very useful resource.	7/16/2020 9:17 AM
12	N/A	7/14/2020 8:47 AM
13	it is kind of clunky and not super easy for searching and going between articles	7/14/2020 7:00 AM
14	The site is not robust, the color scheme is hard on the eyes (light mint color) and I think it could be greatly improved.	7/13/2020 9:26 PM
15	easy to navigate	7/9/2020 12:04 PM
16	Don't use very often	7/9/2020 11:59 AM
17	I have never looked at it.	7/9/2020 7:05 AM
18	Perpetually difficult to access	7/9/2020 1:13 AM
19	Unless I am looking for something specific, I rarely go on the site.	7/8/2020 2:32 PM
20	It would be nice to have the pdf/html links at the top of the individual articles for easy access. I would also like the HTML full text to be on the main page and not have to click for that. Linking to cited JMLA articles in the references on the abstract page would be useful too.	7/8/2020 11:42 AM
21	I think it's just right for the content and what is is being used for.	7/8/2020 10:33 AM
22	I feel like there is an excessive amount of clicking to actually get to the article I want.	7/8/2020 10:32 AM
23	It's not well suited to searching and the OJS layout is a bit old fashioned	7/8/2020 5:23 AM
24	Just 'OK.'	7/8/2020 3:46 AM
25	I'll say the same thing to you that I say to all publishers: appendices and data should be included in the same PDF as the article. There is no reason to make someone have to hunt down data/an appendix in a separate link.	7/7/2020 5:11 PM
26	the green decorative image at the top is incredibly large.	7/7/2020 4:11 PM
27	I found it hard to navigate sometimes, can't find what i need	7/7/2020 3:54 PM
28	I usually only link to the website through the toc when it's emailed to us. I read a couple of articles then, but when I receive the print copy I read it cover to cover.	7/7/2020 3:40 PM
29	N/a	7/7/2020 3:39 PM
30	Never go to the website directly. Always go via the emailed issue links.	7/7/2020 2:54 PM
31	I usually end up in PMC from search results - I just looked at it - the font is off-putting and the article titles of the most recent issue are hard to read	7/7/2020 2:43 PM

32	It seems redundant when JMLA is also available at PMC.	7/7/2020 2:35 PM
33	I like being able to track my training and CEs	7/7/2020 2:05 PM
34	Since I think the entire MLA website is a mess, the JMLA website isn't much better	7/7/2020 1:56 PM
35	I am not really aware of JMLA website, I think of it as the MLA website	7/7/2020 12:54 PM
36	Inaccessible to me	7/7/2020 12:47 PM
37	Search feature works very well. Link from search results goes to journal issue not direct to article. Why?	7/7/2020 12:39 PM
38	It seems a little outdated	7/7/2020 11:48 AM
39	I'm neutral - I don't really use it.	7/7/2020 11:48 AM
40	rarely go there	7/7/2020 10:47 AM
41	Those blue links are hard to read.	7/7/2020 10:42 AM
42	I do not have any particular comment at this time.	7/7/2020 10:40 AM
43	I think today is the first day I have used the JMLA website. It seems easy to navigate, but I have only looked at the current issue.	7/7/2020 10:33 AM
44	Can be cumbersome to use particularly for the submission process.	7/7/2020 10:17 AM
45	Online-First (ahead of print) paper would be helpful	7/7/2020 9:40 AM
46	It's fairly plain, but that makes it easier to find stuff.	7/7/2020 9:23 AM
47	Question 12 is hard to answer -- I frequently want to access supplemental material but it's not available. I was amazed when I learned that the pre-2017 supplemental material related to JMLA articles is not available on the journal site, and that JMLA leaders don't plan to do anything about it because of the cost and human effort. It's really a physician heal thyself situation -- librarians would rake any other journal over the coals for this. It's a breach of trust with the readers who can't access supplemental materials and it's a breach of trust with the authors, who may have assumed that the journal was taking on the responsibility of archiving and making available the supplemental materials they had uploaded.	7/7/2020 8:59 AM
48	n/a	7/7/2020 8:50 AM
49	Old fashioned and cumbersome, but usable	7/7/2020 8:48 AM
50	I've never been on the JMLA website. Didn't know about it.	7/7/2020 8:30 AM
51	It's fine for current issues, but finding previous issue content is more difficult and the search box is not particularly useful.	7/7/2020 7:36 AM
52	The website design and navigation looks outdated and unprofessional.	7/7/2020 7:31 AM
53	None	7/7/2020 7:18 AM
54	Very good. It always shows the trending issues.	7/7/2020 5:10 AM
55	good and should provide free access to ebooks etc.,	7/7/2020 3:20 AM
56	It's very important to get the appendices of surveys, etc.	7/6/2020 11:58 PM
57	a bit cluttered	7/6/2020 8:34 PM
58	The website seems dated.	7/6/2020 7:13 PM
59	I hate all of the clicking through to get to the PDFs of articles. One click please!	7/6/2020 7:09 PM

Q15 Compared with other peer-reviewed journals for health sciences librarians and information professionals, what is the quality of JMLA? [choose one]

Answered: 427   Skipped: 41

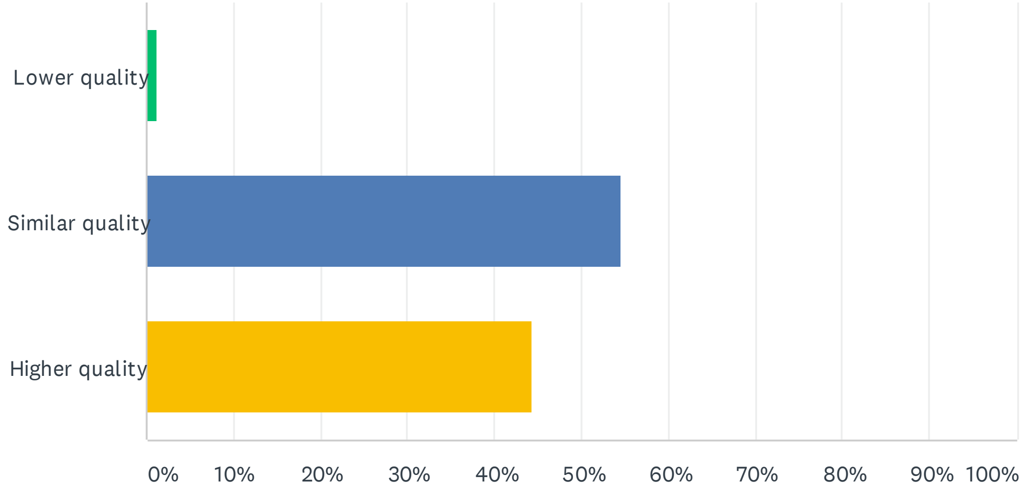

ANSWER CHOICES	RESPONSES	
Lower quality	1.17%	5
Similar quality	54.57%	233
Higher quality	44.26%	189
TOTAL		427

Q16 Please add any other comments about JMLA from a reader's perspective:

Answered: 56 Skipped: 412

#	RESPONSES	DATE
1	A really top outlet - where I'd be happy to publish as a tenure-track information science faculty member - and which is also accessible to colleagues who are working practitioners in libraries.	7/19/2020 9:45 PM
2	It reflects the most important work for our profession. Very helpful	7/19/2020 8:30 PM
3	I miss the more informal "how we did it well" articles. Suspect those stories are available on other platforms that I'm not using.	7/18/2020 10:16 PM
4	thanks! I am glad I have the email alerts because my work days are so busy, I'd miss the content!	7/17/2020 12:49 PM
5	JMLA is a useful resource for providing high quality information. Most issues provide at least one article (if not more) of interest and relevance to my work and interests.	7/16/2020 9:49 PM
6	I normally search for JMLA articles, when I have a library project to work on. I always scan the print issue when it comes in, and always find some interesting articles in it.	7/16/2020 6:07 PM
7	I still enjoy receiving the print copy - so portable - prompts me to read certain articles in depth - and I always know I can get back to other articles online. Keep up the good work!	7/16/2020 2:03 PM
8	Keep up the great work!!	7/16/2020 12:31 PM
9	Read it less now print no longer comes with membership. I'll scan thru the e-mails, but I used to always look at the ToC when I got the print and automatically start reading.	7/16/2020 11:34 AM
10	I appreciate the variety of articles that represent all phases of our profession	7/16/2020 11:20 AM
11	I appreciate JMLA regularity and good standard of publication.	7/16/2020 10:58 AM
12	I already did! But re the data and appendices. I may never access them in an index, but they should be accessible for anyone at anytime. We complain about medical books that remove the References from the end of chapters, do NOT do that to our own. Also, #7: I access both print and online (usually PMC), so why isn't that option available? You all do good, hard, important work. My beef is more with MLA than JMLA, but you're that mouthpiece, so you're a part of it. Keep up the good work, but keep improving. Stay well.	7/16/2020 9:46 AM
13	thanks for a great service	7/16/2020 9:38 AM
14	Thank you for your effort and professionalism.	7/16/2020 9:17 AM
15	JMLA is essential reading for a health sciences librarian! The content and editorial quality are excellent!	7/15/2020 6:48 PM
16	N/A	7/14/2020 8:47 AM
17	I am looking for content that will both support my professional development and assist me in keeping current in my profession. While I don't expect every article to apply to my work environment, I am a Medical Librarian working in a medical facility and this is the Medical Library Association.	7/13/2020 9:26 PM
18	I regard JMLA as the preeminent health sciences libraries journal.	7/13/2020 10:55 AM
19	I am super pleased with the quality of JMLA articles, I think they are the standard to which all Library Science articles should be held.	7/9/2020 4:35 PM
20	organization makes it easy to read	7/9/2020 12:04 PM
21	My one issue is the citation style requirement for in-text and end-of-text bibliographies. I would much prefer (Smith, 1988) to [1], and to have the bibliography organized by 1st author/last name. As an author this is a huge pain if revisions happen; as a researcher, I review the bib, scanning for seminal authors, and in consideration of the original intent for the in-text citation style, I wonder if it wasn't about saving space - no longer a needed element in online publications.	7/9/2020 11:53 AM
22	Distant, not engaged	7/9/2020 1:13 AM
23	JMLA does many things right. Thank you!	7/8/2020 2:35 PM
24	As a hospital librarian, I often find nothing of interest in JMLA...but as a member of a profession, I feel it is important to at least peruse the TOC.	7/8/2020 2:32 PM

25	Keep up the excellent work! Thanks to all volunteers.	7/8/2020 2:10 PM
26	JMLA is the journal for health sciences librarians. I love the fact that it's indexed in PubMed since those outside of librarianship are more likely to discover our work that way. I appreciate that it's open access but does not charge article processing fees. Finally, I feel that the rigor and quality of the work published has consistently improved over the last five or so years.	7/8/2020 5:23 AM
27	As a scientist and librarian, I look for articles that have an 'outward look' on the services for and needs of researchers. Many JMLA contributions--thus, their authors as well--look way too much onto their own profession. Moreover, the vast majority of US article authors/librarians lack any professional medical or scientific education. Thus, much of their activity is irrelevant and could be eliminated, and this pertains a fortiori to many JMLA articles.	7/8/2020 3:46 AM
28	Thank you JMLA editor and editorial staff.	7/7/2020 5:06 PM
29	JMLA is the best! I was so sorry to see SLA's journal go downhill. I am so grateful that JMLA has stayed on top!	7/7/2020 5:03 PM
30	The search text box could work better on the website. I understand if they decide to stop printing it, but I really enjoy the printed editions.	7/7/2020 3:40 PM
31	I am glad to have access to the body of knowledge related to medical librarianship. One suggestion: expand coverage of data science research related to our field.	7/7/2020 3:39 PM
32	I like having these issues indexed in PubMed.	7/7/2020 2:54 PM
33	JMLA is my favorite journal and I love that it is open access! I would never submit an article myself to a journal that is not open access.	7/7/2020 2:35 PM
34	I actually don't look at other peer-reviewed journals for librarians so my previous answer isn't very accurate.	7/7/2020 2:05 PM
35	Would like more information on what is new. Databases, & comparing items. Maybe a spot lights on items or things you need to know section. To know what is out there. Sometimes I feel lost or not informed when items are mentioned that I never heard about.	7/7/2020 1:32 PM
36	I read what interests me, not always the same article type	7/7/2020 12:54 PM
37	Thank you for your work to provide a great journal of medical librarianship!	7/7/2020 12:39 PM
38	JMLA is indispensable!	7/7/2020 11:48 AM
39	Honestly - keep the content a bit lighter? While I recognize the importance of scholarly work in our field, I'd like to see more coverage of day-to-day issues faced by Medical Librarians, rather than in-depth research studies. I'd like to see: A review of predatory publishing; a review of read and publish models, a review of libraries & COVID_19. I certainly appreciate all the work that goes in to detailed research studies, but my staff and I don't have time to participate in such studies. We are not required to publish (we're not faculty track). I'd love to see more practical information covered. Thanks for all the hard work, and thanks for asking for our input.	7/7/2020 10:53 AM
40	It would be great if there were some more high quality research studies for advanced medical library professionals. It might attract readers not limited to information professionals.	7/7/2020 10:40 AM
41	I have always appreciated the JMLA, receiving the print copies regularly when I was a younger librarian. Today I mostly rely on topic searches in LISTA to find the library literature I read, but now that I have discovered the JMLA website I may go there on a regular basis.	7/7/2020 10:33 AM
42	Question 14 worries me. There are topics that *only* health sciences librarians engage in research about, and there are topics for which JMLA is competing with medical informatics (to name only one other field). Many peer-reviewed journals for LIS are of very low research quality indeed, even if they serve a valuable community-building and tip-exchanging function for the field (tip-exchanging, in particular, is something I thought was killed by the Web in approximately 1993. I was wrong). Don't compare yourselves with only LIS publications! Compare yourselves with other Web of Science ranked publications in information science and medicine!	7/7/2020 10:14 AM
43	Please put the pre-2017 supplemental materials on the current JMLA site or into some kind of searchable data repository -- Figshare? OSF?	7/7/2020 8:59 AM
44	n/a	7/7/2020 8:50 AM

45	It is on the profession to provide the studies and data that would heighten the quality of JMLA. As it stands, it is a fine journal for a perception of librarianship held mostly by the public. We should see ourselves more as research practitioners. JMLA will evolve when the profession evolves. Until then, carry on, and thank you for all you do.	7/7/2020 8:49 AM
46	The work to improve the openness and transparency of our work is much appreciated.	7/7/2020 8:48 AM
47	I'd like to have a newsletter highlighting some articles sent more often than once a quarter.	7/7/2020 8:40 AM
48	I don't read the entire journal since it's online. If I had print, I would be more apt to read in its entirety. Not a fan of e-reading.	7/7/2020 8:30 AM
49	I find the journal content and website very disappointing.	7/7/2020 7:31 AM
50	I find JMLA articles to be highly theoretical, which makes relating the content to what I do challenging. I am excited to read the new Knowledge Synthesis articles and I have always enjoyed the Case Reports, Case Studies, and Virtual Project submissions.	7/7/2020 7:20 AM
51	I think most of the articles are for the benefit of the academic librarians CVs - JMLA gives them a place to publish. There's not much of practical use for the hospital librarian.	7/7/2020 7:18 AM
52	JMLA it is a mark.	7/7/2020 5:10 AM
53	very good	7/7/2020 3:20 AM
54	Sometimes i like to participate in the seminars and workshops of JMLA but its very expensive for me. Iran Sanctioned by America and Iran's currency hits lowest value ever against the dollar.	7/7/2020 12:59 AM
55	I appreciate that the articles are open access.	7/6/2020 7:20 PM
56	I wish the focus was a little broader	7/6/2020 7:09 PM
